# Mental health profiles of 15-year-old adolescents in the Nordic Countries from 2002 to 2022: person-oriented analyses

**DOI:** 10.1186/s12889-024-19822-x

**Published:** 2024-08-30

**Authors:** Charli Eriksson, Håkan Stattin

**Affiliations:** 1 Department of Learning, Informatics, Management and Ethics, Karolinska Institute, SE-17177 Stockholm, Sweden; 2 Department of Psychology, Uppsala University, Box 1225, 75142 Uppsala, Sweden

**Keywords:** Mental health, Psychological symptoms, Somatic symptoms, Adolescents, Cluster analysis, Dual-factor model

## Abstract

**Background:**

Studies of time trends in Nordic adolescents' mental health have often relied on analyses of adolescents’ psychosomatic symptoms. In this study, we examine adolescents' self-reports on mental health in the context of the dual factor model, which encompasses both overt manifestations of mental health symptoms and subjective perception of one's health status.

**Method:**

The objective of this study was to employ a person-oriented approach utilizing cluster analysis to discern time trends in mental health profiles of Nordic adolescents, using their psychosomatic complaints and their perception of their overall health as cluster variables. The resulting health profiles were then subjected to a comparative analysis with regard to different measures of psychological and social adjustment. The mental health profiles were based on data from the Health Behaviour in School-aged Children (HBSC) survey, which was conducted among almost 50000 15-year-olds in five Nordic countries (Denmark, Finland, Iceland, Norway, and Sweden) between 2002 and 2022.

**Results:**

Mental health profiles exhibiting comparable content were observed in all Nordic countries, including profiles indicating adequate health, perceived good health, perceived poor health, high psychosomatic complaints, and dual health problems (defined as both high psychosomatic complaints and perceived poor health). These health profiles showed similar trends over time in the Nordic countries. Significant gender differences were observed. In 2002, adequate health was the dominant profile for both sexes. After 20 years, however, the high psychosomatic profile became the most common profile among girls. Among the three risk profiles, namely perceived poor health, high psychosomatic complaints and dual health problems, adolescents in the dual problems profile had the most psychological and social adjustment problems.

**Conclusions:**

The comparatively lower incidence of adjustment problems among adolescents in the high psychosomatic profile relative to the dual mental health group challenges the prevailing view that there has been a sharp increase in mental health problems among Nordic adolescents. This view was largely based on the observed rise in psychosomatic symptoms. Indeed, there was a doubling in the proportion of adolescents in the high psychosomatic complaints profile between 2002 and 2022. This increase was considerably more pronounced than that observed for the dual health problems profile which exhibited most problems.

## Introduction

There is cause for concern regarding the mental health of adolescents in the Nordic countries. A number of reports have indicated a significant decline in mental health among adolescent populations in high-income countries between the 1950s and the present day [1–5]. This decline has been particularly evident in the Nordic countries. The reports have primarily based this conclusion on the marked increase in psychosomatic symptoms over time. This raises the question of whether different mental health indicators show similar time trends in the Nordic countries. The aim of this study is to examine the combined long-term trends of two mental health indicators—psychosomatic complaints and perceived overall health—among adolescents in Denmark, Finland, Iceland, Norway, and Sweden. Nationally representative samples of adolescents participating in the Health Behaviour in School-aged Children (HBSC) study in these countries between 2002 and 2022 [6] were used. The dual-factor model [7–9] was employed as the analytical framework, with cluster analysis employed to examine adolescents' psychosomatic complaints and their perceived health in a single model in these time-trend analyses.

Repeated cross-sectional surveys, such as the HBSC survey [10], provide insight into temporal trends in adolescent psychosomatic symptoms [11]. A meta-analysis of 21 studies found a slight increase in self-reported psychosomatic complaints among adolescents between the 1980s and 2000s [5], but subgroup analysis supported a significant increasing trend only in Northern Europe. A study in four Nordic countries that conducted a trend analysis of data collected in the HBSC study between 1994 and 2014 [12] found an increasing rate of psychosomatic complaints problems among adolescents. The study showed that Finland, together with Sweden, had the highest increase in psychosomatic complaints among grade 9 adolescents, especially among girls. In another study analysing trends in psychosomatic complaints using data collected between 1985 and 2005 among students in grades 5, 7 and 9 in Sweden, Hagquist [13] found significantly higher rates of psychosomatic complaints in 2005 compared with 1985 among adolescents in grade 9, again especially among girls, while the rates were almost unchanged among boys and girls in grades 5 and 7. A Swedish HBSC study over three decades [14] found that the increase in psychosomatic complaints was greatest among students who reported frequent and co-occurring psychosomatic complaints. A Norwegian HBSC study of psychological and somatic health complaints showed that these followed somewhat different trajectories, but the mean scores for both types of health complaints appeared to increase over a 20-year period [15]. A Danish study reported a higher prevalence of emotional complaints in higher socioeconomic classes [16]. These studies, as well as surveys of regional samples in the Nordic countries [17–23], support the conclusion that there has been an increase in psychosomatic complaints in recent decades among both middle-aged boys and girls, especially among 15-year-old girls, but not much among younger adolescents.

A comparable increase in adolescents' perceptions of their overall health [24] or, for that matter, in lower life satisfaction [25] is not observed. A trend analysis of excellent perceived overall health for Nordic adolescents showed a slight improvement between 2002 and 2006, but a stable trend was found in subsequent periods up to 2014 [24]. Eriksson and Stattin [26] reported some changes in perceived overall health among Swedish 15-year-olds between 2002 and 2018. These changes over these survey years had very small effect sizes. The Swedish Public Health Agency [27] reported results for adolescents' perceptions of very good health for the survey years 2002 to 2022. They found a stable trend over this period, with a smaller dip in 2014. A longitudinal study in Norway followed adolescents aged 13–19 years for 4 years [28]. They found high stability for the measure of perceived overall health, with 59 per cent of participants reporting the same response option at both time points, and there was a small, insignificant decrease in this measure of perceived overall health over the four years. In short, while there appears to have been a more substantial increase in psychosomatic complaints among Nordic adolescents in recent decades, these adolescents' perceptions of their overall health do not appear to have changed much over the same period.

### A dual-factor model

The overall conclusion from studies of time trends in psychosomatic complaints and perceived health status among adolescents in the Nordic countries is that there are marked differences between these time trends. To get a more complete picture, an overarching analytical model is needed that combines these two indicators of mental health. The traditional model of mental health in the literature uses a single dimension, ranging from good mental health (or absence of complaints) to mental health problems (manifested by a high prevalence of psychopathological symptoms). The dual-factor models of mental health avoid the limitations of a single dimension. They propose that mental health encompasses two interrelated but distinct dimensions: psychopathology and well-being [7, 29–31]. Applied to the present study, with non-clinical indicators of problematic mental health, the dual-factor model can be described as identifying the typical subgroups or profiles that encompass both psychosomatic complaints and self-perceived health status in a population of young people. The World Health Organization [32] definition of health status includes "a state of complete physical, mental and social well-being and not merely the absence of disease or infirmity", i.e. not only the presence or absence of disease, but also people's perceptions of their physical, mental and social well-being are required. This is in line with the integration of young people's perceptions of their overall health status and their psychosomatic complaints in the present study.

The usual argument for a dual-factor approach is that the absence of psychopathology does not necessarily imply the presence of well-being [9]. However, one can argue from a different perspective. The increase in psychosomatic complaints among adolescents in recent decades, particularly in the northern countries, has been interpreted in many publications as a strong indication of a deteriorating trend in mental health among young people [14, 18, 19, 33, 34]. A plethora of studies have proffered disparate explanations for this increase over time, rendering a comprehensive review impractical [35–41]. But the presence of psychosomatic complaints among young people does not necessarily indicate that these young people are at risk of more severe psychopathology or adjustment problems. It may depend on their overall perception of their health status. If these young people's overall health is perceived as good, they may still be able to grow and develop. However, if their psychosomatic complaints coincide with a judgement that their overall health status is poor, they may be at risk of developing adjustment problems and more serious mental health problems. It seems important to distinguish between psychosomatic problems that occur with or without perceived poor overall health. To illustrate, the Swedish Agency for Youth and Civil Society (MUCF) is a government agency tasked with monitoring the living conditions of young people. According to MUCF's 2024 report, 56% of young people aged 16–24 in Sweden reported experiencing anxiety or worry in 2022. The highest proportion of mental health symptoms was reported by girls with a Swedish background, with 76% reporting such symptoms. These high rates of mental health problems are indeed a cause for concern. However, in the same study, 77% of young people rated their overall health as good. This significant discrepancy between mental health symptoms and perceived overall health raises questions about how to conceptualize young people's mental health status.

There is previous support for the hypothesis that the consequences of high psychosomatic complaints on adjustment depend on individuals’ overall perception of their health status. The dual-factor approach, with the two mental health indicators perception of overall health and psychosomatic complaints, was previously employed in a study of Swedish adolescents. The study included 11-, 13-, and 15-year-olds in the HBSC data collection conducted in 2017/18 [42]. Cluster analyses yielded four distinct mental health profiles: “perceived good health”, “perceived poor health”, “high psychosomatic symptoms, and “poor mental health”, which encompasses both perceived poor health and high psychosomatic symptoms. The four mental health profiles were observed in all age groups. Positive self-perception, positive school experiences, and perceived social support from family and friends were compared between the four mental health profiles for the 15-year-olds. The adolescents exhibiting the poorest adjustment were those presenting high levels of psychic and somatic symptoms in conjunction with low levels of perceived overall health. This evidence lends support to the assumption that when adolescents experience mental health issues, such as psychosomatic disorders, in conjunction with a negative perception of their overall health, it should be regarded as an indicator of more severe mental health concerns than experiencing psychosomatic symptoms only.

A second study [26] examined time trends in the mental health profiles of Swedish 15-year-olds between 2002 and 2018, also with cluster analysis of psychosomatic complaints and perception of overall health. The same four factors were found in all survey years, but the time trend results pertain solely to Swedish 15-year-olds. The present study builds upon these findings by examining time trends over 20 years in the mental health profiles of 15-year-old adolescents from the Nordic countries of Denmark, Finland, Iceland, Norway, and Sweden. The aim is to ascertain to what extent the secular trends in the mental health profiles of Nordic adolescents align.

### Current study

The current study analyses time trends from 2002 to 2022 for psychosomatic problems and perceived overall health status among 15-year-old adolescents in five Nordic countries. We use cluster analysis to identify naturally occurring cluster constellations over this 20-year period. The aim is to investigate whether the mental health profiles identified among adolescents in the different countries represent a general developmental pattern in the Nordic region. As the gender gap in adolescent mental health seems to be omnipresent across cultures, with girls having worse mental health [43], the time trends in the mental health profiles are compared separately for boys and girls in the analyses.

Finally, we examine which mental health profiles are particularly associated with psychological and social adjustment problems, including feelings of stress, loneliness, poor communication with parents, low school adjustment, irregular health habits, and use of medication for psychological and somatic reasons. Additionally, the mental health profiles were compared with a measure of family wealth. Furthermore, a robustness analysis was conducted to examine whether similar mental health profiles would emerge when perceived overall health was exchanged with a well-being measure, namely life satisfaction.

## Methods

### Sample

The HBSC study employs repeated cross-sectional surveys to collect data from adolescents in the five Nordic countries every four years, spanning the period from 2002 to 2022. The HBSC study is an international, collaborative, cross-national survey conducted by the World Health Organization with the overarching objective of enhancing comprehension of the health behaviors exhibited by young people aged 11, 13, and 15. This study encompasses HBSC data on 15-year-olds from five countries, with surveys conducted in 2002, 2006, 2010, 2014, 2018, and 2022.

In Denmark, Finland, Norway and Sweden, samples were drawn randomly by cluster sampling within strata defined by geographical area in order to obtain nationally representative data sets. School classes were used as sampling units and stratification was proportional. The recommended national sample size per class was 1500, and the mean age should be 15.5 years, with 90% of the sample falling within ± 6 months of the mean age. Iceland invited all schools in the country from 2010 onwards and therefore had a larger sample than the other countries. Table 1 shows the total participation for each survey year and for each country. A total of 48948 15-year-olds participated in all survey years.

**Table 1:** Participation in the five Nordic countries between 2002 to 2022 (*N* = 48948)

	2002	2006	2010	2014	2018	2022
Total sample						
Denmark	1350	1515	1215	1249	919	1642
Finland	1724	1568	2062	1951	1135	993
Iceland	0	1865	3636	3288	2210	2979
Norway	1600	1494	1319	905	858	987
Sweden	1198	1508	2041	2683	1621	1433

Students completed the internationally standardized questionnaire after being instructed by their teacher. Verbal and written information about the confidentiality of their answers was provided, and participation was confidential and voluntary. Printed questionnaires were used in the first data collections, but web-based questionnaires were introduced in Norway in 2010 (both paper-and-pencil and computer and from 2018 only web-based), in Denmark from 2014 only web-based, in Finland from 2018 only web-based, in Iceland both since 2018 and in Sweden both methods were used in 2022.

A standardised international research protocol was followed to ensure consistency in survey instruments, data collection and processing procedures. Schools or classes that refused to participate, and students who were absent on the day of the survey, were the main sources of non-response and were not followed up.

The HBSC Data Management Centre at the University of Bergen, Norway, checked the quality of the data collected, performed appropriate cleaning of the data and merged the national data sets into a Nordic data file. Detailed information on the study can be found on the HBSC website [44]. The methodology for data collection is described in the HBSC protocol [45], which requires consistency in sampling plans, survey instruments and data collection.

### Measures

In this study, non-clinical indicators of problematic mental health were measured. We do not include measures of psychopathology and psychiatric diagnoses (such as depression, anxiety, suicidal ideation, psychosis, etc.). The clustering variables include two measures of mental health problems. One is the HBSC Complaint Checklist (HBSC-SCL), also known as psychosomatic symptoms, and the other is perceived overall health, also known as self-rated health.

Psychosomatic complaints were measured by the question "In the last 6 months, how often have you had the following…" and the eight items: 'headache', 'stomachache', 'backache', 'feeling low', 'irritability or bad temper', 'feeling nervous', 'difficulty falling asleep' and 'feeling dizzy'. The response categories are: 'about every day', 'more than once a week', 'about every week', 'about every month' and 'rarely or never', reverse coded. This question has been included in the HBSC questionnaire since 1985/86 [45]. A recent study of 46 countries examined the dimensionality of the HBSC Complaint Scale using exploratory graph analysis [46] and found unidimensionality in 16 countries and negligible deviation from the unidimensional structure in most other countries. The HBSC Complaint Checklist has been shown to have acceptable test–retest reliability and internal consistency [47, 48]. Cronbach's alpha was 0.84.

Perceived overall health was measured by the single item 'Would you say your health is …'. Participants were asked to rate their overall health by selecting one of the response categories (1) poor, (2) fair, (3) good and (4) excellent [45], coded inversely to tap perceived overall unhealth. This item is an attempt to understand how adolescents perceive their own overall health status without asking for specificity.

To determine which mental health profiles could be considered risk profiles, we used seven indicators of psychological and social adjustment. They were measured in all survey years except for feeling stressed, feeling lonely, and taking medication, which were measured only in 2022.

Feeling stressed was a two-item measure: “Felt unable to control important things in life” and “Felt difficulties were piling up so high that you could not overcome them” [45]. The response scale ranged from (1) never to (5) very often. The correlation between the two variables was 0.51, *p* < 0.001.

Loneliness was a single item measure [49]. The individuals were asked if they had felt lonely the past 12 months. The response scale ranged from (1) never to (5) always. Loneliness, measured with this item, was found to be a strong indicator of low mental well-being and low self-esteem in all Nordic countries [50]

Regular health habits was a scale measuring whether the adolescents had regular health habits in everyday life: brushing their teeth ((1) never to (5) more than once a day), being physically active for at least 60 min in the last 7 days ((0) 0 days to (7) 7 days), eating breakfast on weekdays ((1) never to (6) five days) and eating fruit ((1) never to (6) more than once a day) [45]. The measure was an attempt to describe the extent of a person's regular health behavior in everyday life. It was not anticipated that there would be a high degree of correlation between individual items (the inter-item correlation was 0.17).

Parental communication was a measure of the ease of communicating with mothers and fathers, measured by asking respondents how easy it was for them to talk to their father or mother about things that really bothered them. The five response options were (1) don't have or see that person, (2) very difficult, (3) difficult, (2) easy and (5) very easy [45]. Adolescents who answered that they had no contact with the specific parent were assigned a missing value (5.3% for communication with fathers and 1.6% for communication with mothers). The correlation between perceived ease of communication with fathers and mothers was 0.57 (*p* < 0.001) across countries and survey years.

Liking school was a single item measure: I like school, with a response scale ranging from (1) not at all to (4) a lot [45].

Participants were asked if they had taken medication in the last month for the following problems: headache, stomachache, backache, sleeping problems or/and nervousness [45]. This question was only asked of adolescents in Denmark, Finland and Sweden in the 2022 survey year.

Perceived family wealth was measured using a single measure of family socio-economic status. The item was "How well off do you think your family is?", with the response scale ranging from (1) not at all well off to (5) very well off [45].

In a robustness assessment of the mental health profiles, we employed a measure of the individuals’ life satisfaction. This was a single item, the Cantril Ladder, where adolescents rated their life satisfaction on a scale from 0 (indicating the worst possible life) to 10 (indicating the best possible life) [45, 51].

Sex was reported via the item "Are you a boy or a girl?" and coded as (0) male or (1) female.

### Analytic methods

Cluster analysis was used to identify the naturally occurring clusters or profiles of psychosomatic complaints and perceived poor health in the five national samples. First, the eight-item psychosomatic complaints scale was used in cluster analysis together with the self-reported overall health item. Both measures were standardised. Hierarchical cluster analysis (Ward's method) was then used to determine the number of clusters. A lower limit of 67% was set for the number of clusters selected to explain the total error sums of squares [52]. For all countries except Norway, we found four-cluster solutions (Error sums of squares: Finland: 71.8%; Denmark: 71.6%; Iceland: 68.3%; Norway: 66%; Sweden 73%; all five countries combined: 71.9%), similar to previous studies of Swedish adolescents [26, 42]. However, to distinguish more diverse mental health profiles to be compared between countries, we decided to test a five-cluster solution. This five-cluster solution was applied to all five countries. Knowing the number of clusters, non-hierarchical cluster analysis, K-means clustering, were used to arrive at the final cluster solutions.

The second step was to compare the mental health profiles that emerged over the six survey years (a) between the five Nordic countries, (b) between boys and girls, and (c) between combinations of survey years, countries and gender. We used the adjusted standardized residuals in cross-tabulations to estimate differences between countries, survey years and sex. The adjusted standardized residuals in a contingency table can be roughly interpreted as standard normally distributed, and values greater than or equal to 3.29 or lower than or equal to -3.29 indicate that the cell deviates significantly from the null hypothesis at the 0.001 level. Finally, we employed crosstabulations to examine the differences between survey years in the five mental health profiles, separately for boys and girls.

Then, in a third step, the mental health profiles were compared for psychological and social adjustment characteristics – feeling stressed, feeling lonely, regular health habits, parental relationships, school adjustment, taking medication and perceived family affluence. There are few examples in the literature where the relationships between psychosomatic complaints and perceived overall health, on the one hand, and different measures of psychological and social adjustment, on the other, have been included in the same study. Therefore, we started by providing information on the relationships between psychosomatic complaints and perceived overall health on the one hand, and the measures of psychological and social adjustment on the other, and then compared the mental health profiles for each of these psychological and social characteristics using one-way ANOVAs.

The final step was an analysis of the robustness of the cluster analysis by using life satisfaction instead of perceived overall health in the cluster analysis. An indication of robustness would be if similar profiles were found when using life satisfaction as when using perceived overall health.

## Results

The time trends were initially examined separately for the two measures of perceptions of low overall health and psychosomatic complaints across countries and sexes (Table 2). In addition, perceptions of low life satisfaction were included in the study because this measure will be used in subsequent investigations of the robustness of the mental health profiles. The results indicate that there are virtually no trends in perceptions of low overall health, a very small increase in the final year of the survey for low life satisfaction, but a larger incremental increase over the survey years for psychosomatic complaints. This corroborates previous studies that have demonstrated a lack of change over recent decades in perceptions of overall health and life satisfaction, while revealing an increase over time for psychosomatic complaints.

**Table 2:** Trends in perceived low overall health, low life satisfaction and psychosomatic complaints for the five countries

	Perceived low overall health	Low life satisfaction	Psychosomatic complaints
2002	-0.01^a^	-0.01^b^	-0.27^e^
2006	-0.01^a^	-0.08^c^	-0.14^d^
2010	-0.00^a^	-0.06^c^	-0.13^d^
2014	-0.01^a^	0.02^b^	-0.00^c^
2018	-0.00^a^	0.01^b^	0.23^b^
2022	0.02^a^	0.13^a^	0.32^a^

The measures are standardized

Different superscripts ^a b c^ represent significant differences (*p* < .05) between the four cluster groups employing Student Neuman Keul’s post-hoc test

Perceived low overall health: F (5, 48875) = 0.74, *p* = .592, eta^2^ = 0.00

Low life satisfaction: F (5, 48395) = 50.50, *p* < .001, eta^2^ = 0.01

Psychosomatic complaints: F (5, 48810) = 390.33, *p* < .001, eta^2^ = 0.04

The research question is whether similar types of mental health profiles can be found in the five Nordic countries. Table 3 shows the profiles obtained when cluster analyses were performed separately for each of the five countries, aggregated over the survey year. The following guidelines were used for interpreting the clusters: a low standardized centroid value is < -0.70, an average value is between -0.70 and 0.70, and a high value is > 0.70. Table 3 indicates that the majority of mental health profiles observed in the five Nordic countries are similar. These profiles were labelled "adequate mental health," "perceived good health," "perceived poor health," "high psychosomatic complaints," and "dual health problems" (both high psychosomatic complaints and high perceived poor health). In addition to the similarities observed in the profiles themselves, the proportions of adolescents in each cluster are also comparable across countries, with a few exceptions. The profile labeled "adequate mental health" had the highest proportion of individuals in all countries, followed by "perceived good health" and "high psychosomatic complaints." The profiles labeled "perceived poor health" and "dual health problems" had the lowest percentages.

**Table 3:** Cluster analysis of mental health profiles for each the five Nordic countries

	Adequate mental health	Perceived good health	Perceived poor health	High psychosomatic complaints	Dual health problems
Denmark ^1^	-0.62	-0.62	0.21	1.40	1.77
Perceived overall unhealthy	0.18	-1.25	0.77	-0.12	1.82
N	2492	2131	1585	1032	622
%	31.7	27.1	20.2	13.1	7.9
Finland^2^					
Psychosomatic complaints	-0.56	-0.62	-0.14	1.02	1.72
Perceived overall unhealthy	0.10	-1.41	1.69	-0.03	1.86
N	3641	2034	825	2267	654
%	38.6	21.6	8.8	24.1	6.9
Iceland^3^					
Psychosomatic complaints	-0.55	-0.68	-0.16	1.07	1.61
Perceived overall unhealth	0.02	-1.32	1.46	-0.23	1.66
N	5165	3035	1545	2771	1372
%	37.2	21.9	11.1	20	9.9
Norway^4^					
Psychosomatic complaints	-0.40	-0.57	-0.26	1.38	1.69
Perceived overall unhealth	0.17	-1.12	1.61	-0.19	1.79
N	2544	2127	815	1189	480
%	35.6	29.7	11.4	16.6	6.7
Sweden^5^					
Psychosomatic complaints	0.43	-0.66	-0.16	1.18	1.62
Perceived overall unhealth	0.27	-1.16	1.81	0.00	1.95
N	3692	3147	705	2222	704
%	35.3	30.1	6.7	21.2	6.7

Low value is < -0.70, Average value is between -0.70 and 0.70, High value is > 0.70

Data were not collected for Iceland in survey year 2002

Explained sums of squares: ^1^ 78.9%, ^2^ 78.4%, ^3^ 76.3%, ^4^ 75.4%, ^5^ 78.3%

With similar mental health configurations for each country, a cluster analysis was carried out including all participants in all five countries and all six survey years. The results of this cluster analysis are shown in Table 4. The two clusters of 15-year-olds with positive mental health, labelled "adequate mental health" and "perceived good health," included almost two-thirds of the individuals. Of the three clusters with negative mental health, the most common cluster was "high psychosomatic complaints." With the same cluster solution for all individuals and common centroids, it is now possible to make direct comparisons between countries, survey years, and between boys and girls. The bottom part of Table 4 shows the sex differences in the five profiles when the data are aggregated across countries and survey years. A crosstabulation was performed between gender and mental health profiles, and the adjusted standardized residuals were examined.

**Table 4:** Cluster analysis of mental health profiles covering countries and survey years combined (*N* = 48,796)

	Adequate mental health	Perceived good health	Perceived poor health	High psychosomatic complaints	Dual health problems
Psychosomatic complaints	-0.51	-0.63	-0.17	1.13	1.69
Perceived overall unhealth	0.13	-1.25	1.62	-0.08	1.79
N	17663	12534	4700	10139	3760
%	36.2	25.7	9.6	20.8	7.7
Boys^1^	39.2	33.6	10	13	4.2
Girls	33.5^ l^	18.1^ l^	9.2	28.2^ h^	10.9^ h^

Low value is < -0.70, average value is between -0.70 and 0.70, high value is > 0.70

The five clusters explain 79.2 percent of the total error sums of squares

^h^ = significantly high value and ^l^ = significantly low value of the adjusted standardized residuals

^1^Differences between sexes: χ^2^(*N* = 48521, *df* = 4) = 3320.57, *p* < .001, Cramer’s V = 0.26

### Overall sex differences

A high adjusted standardized residual indicates that the mental health profiles are significantly overrepresented by boys, whereas low values indicate that these profiles are significantly overrepresented by girls. As illustrated in Table 4, a significantly greater proportion of boys than girls belonged to the adequate mental health cluster. Almost twice as many boys as girls belonged to the perceived good health profile, while more than twice as many girls as boys belonged to the high psychosomatic complaints profile and almost three times as many girls as boys belonged to the dual health problems profile. It is evident that pronounced good mental health is more prevalent among boys, while pronounced mental health problems are more common among girls than among boys. There are no gender differences for the profile of perceived poor health. The cross-tabulation of girls' and boys' mental health profiles demonstrated a significant effect, with the value of Cramer's V (0.26) indicating that the differences between the sexes had a medium effect size.

Finally, we sought to determine whether the observed gender differences in the five mental health profiles varied across countries. An analysis of variance (ANOVA) was conducted to examine the interaction between sex and country, as well as the effect of sex on mental health profiles. The results indicated a non-significant interaction effect (χ^2^(N = 24763, df = 4) = 9.44, p = 0.051, Cramer's V = 0.02), suggesting that the sex differences observed in the mental health profiles between countries were comparable to those within countries.

With the same cluster solution for all individuals and with common centroids, it is now also possible to make direct comparisons between countries and survey years. These comparisons are presented in Table 5.

**Table 5:** Mental health profiles: percentage of respondents in each country and year surveyed

	Adequate mental health	Perceived good mental health	Perceived poor health	High psychosomatic complaints	Dual health problems
Country differences ^1^					
Denmark	42.5^ h^	28.1^ h^	11.5^ h^	12.1^ l^	5.8^ l^
Finland	41.2^ h^	21.1^ l^	8.8^ l^	22^ h^	6.9
Iceland	34.7	21.9^ l^	10.1	22.5^ h^	10.9^ h^
Norway	35.6	31.4^ h^	12.9^ h^	14.9^ l^	5.2^ l^
Sweden	29.4^ l^	29.2^ h^	6.1^ l^	28^ h^	7.3
Differences between survey years ^2^					
2002	41^ h^	27.2	12.8^ h^	13.6^ l^	5.4^ l^
2006	39.6^ h^	25.3	10.9^ h^	17.7^ l^	6.7
2010	39.3^h^	26.4	10.5^ h^	17.2^ l^	6,5^ l^
2014	36.9	24.5^ l^	9	21.9^ h^	7.8
2018	32.5^ l^	24.4	9.1	26.2^ h^	7.8
2022	27.5^ l^	25.7	6.1^ l^	28.8^ h^	11.8^ h^

^h^ = significantly high value and ^l^ = significantly low value of the adjusted standardized residuals

^1^Differences between countries: χ^2^(*N* = 48796, *df* = 16) = 1822.79, *p* < .001, Cramer’s V = 0.10

^2^Differences between survey years: χ^2^(*N* = 48796, *df* = 20) = 1350.05, *p* < .001, Cramer’s V = 0.08

### Country differences

As illustrated in the upper section of Table 5, there were significant differences between the mental health profiles for the countries under examination. However, the effect size was relatively modest (Cramer's V = 0.10), indicating that there were no substantial differences in mental health profiles between the Nordic countries. Iceland and Sweden exhibited the lowest percentages of positive mental health—adequate mental health and perceived good health. Iceland exhibited the highest prevalence of individuals presenting with a dual health problem profile, while Sweden exhibited the highest prevalence of individuals presenting with high psychosomatic complaints.

### Differences between survey years

If there is a general increase in a mental health profile over time, we would expect to find significantly low values of the adjusted standardized residuals in the early years of the survey, followed by significantly high residuals in the final years of the survey. Conversely, the opposite would be the case for declines in a mental health profile over time. Such general declines were observed for the adequate mental health and perceived poor health profiles, and increases were noted for the high psychosomatic complaints profile and, to some extent, for the dual health problems profile. However, these differences between survey years can be considered small. Cramer's V was 0.08, which is a small effect size. Nevertheless, a comparison between 2002 and 2022 revealed a 30% decrease in the percentage of adolescents with an adequate health profile. The proportion of adolescents in the cluster with high psychosomatic complaints increased by twofold between 2002 and 2022 (from 13.6 to 28.8 percent), and the profile with dual health problems also increased by twofold between these years (from 5.4 to 11.8 percent).

### Changes in the five mental health profiles over the survey years for each country

A main objective of this study was to examine whether the developmental trends for the five mental health profiles are similar across countries. To this end, for each country, we calculated the proportions of adolescents belonging to these profiles in each of the six survey years. The results revealed a high degree of similarity. Table 6 provides a simplified overview and shows the percentages for the first and last survey years, 2002 and 2022. To test for increases or decreases in the profiles over the twenty-year period, we employed the adjusted standardized residuals. A high value (h) for these residuals indicates a significant increase between 2002 and 2022, while a low value (l) indicates a significant decrease. The table demonstrates a striking similarity in the increases and decreases in mental health profiles across countries. There was a significant decrease in the proportion of people with an adequate mental health profile in all five countries. While there were some differences in the perceived good mental health profile, three countries demonstrated no significant change in this profile between 2002 and 2022. Iceland, however, exhibited a significant increase, while Sweden demonstrated a significant decrease over the course of the 20-year period. Four countries demonstrated a significant decrease in the perceived poor health profile, but no significant differences were found for Finland. A significant increase in severe psychosomatic complaints was observed in all countries, with the exception of Sweden. Overall, it can be concluded that there have been remarkably similar increases and decreases in mental health profiles in the Nordic countries.

**Table 6:** Each country: Percentage of respondents belonging to the mental health profiles in 2002 and 2022

	Adequate mental health	Perceived good mental health	Perceived poor health	High psychosomatic complaints	Dual health problems	Cramer’s V
Denmark:						
2002	47.9	27.6	13.6	7.3	3.6	
2022	34.9^ l^	27.4	7.9^ l^	19^ h^	10.7^ h^	0.26
Finland:						
2002	42.6	25.1	8.7	18	5.6	
2022	32.4^ l^	20.1	6.5	27.6^ h^	13.4^ h^	0.19
Iceland:						
2006	40.2	18.9	11.4	19.9	9.5	
2022	24^ l^	25.7^ h^	6.1 l	30.2^ h^	14^ h^	0.22
Norway:						
2002	41.3	25.6	19.1	9.5	4.6	
2022	27.3^ l^	30.9	8.2^ l^	24.2^ h^	9.4^ h^	0.28
Sweden:						
2002	30.5	32	9.2	20	8.3	
2022	22.8^ l^	24.2^ l^	2.5^ l^	41.2^ h^	9.3	0.26

^h^ = significantly high value and ^l^ = significantly low value of the adjusted standardized residuals

### Sex differences in the separate mental health profiles across time

**Fig. 1:**
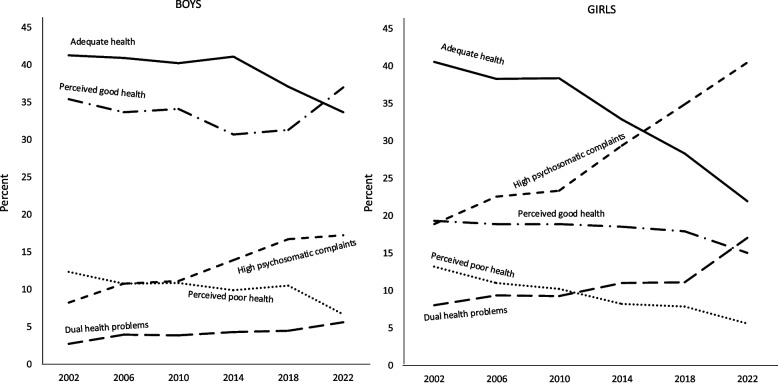
Changes over time in the five profiles of mental health among boys and girls

Given the limited differences in time trends for the mental health profile between countries, we sought to determine whether the mental health profiles of boys and girls evolved differently from the survey year 2000 to 2022. The details are presented in Table 7 and graphically illustrated in Fig. 1. The mental health profiles for boys exhibited relatively minor changes over time, with a modest effect size (Cramer's V = 0.06). As illustrated in the upper section of Table 7, the only consistent increase observed in boys' mental health profiles was in psychosomatic problems. Conversely, there were no consistent decreases across the survey years, with the exception of perceived poor health. In contrast, girls exhibited a greater number of changes in their mental health profiles over time, although the effect sizes remained relatively small (Cramer's V = 0.11). As illustrated in the lower section of Table 7, there were more general decreases over the survey years for the profiles of adequate mental health and perceived poor health, while there were increases for high psychosomatic complaints and dual health problems for girls.

**Table 7:** Differences between survey years in the five mental health profiles, separately for boys and girls

	Adequate mental health	Perceived good mental health	Perceived poor health	High psychosomatic complaints	Dual health problems
Boys:					
2002	41.3	35.4	12.3^ h^	8.2^ l^	2.7^ l^
2006	40.9	33.7	10.7	10.7^ l^	3.9
2010	40.2	34.1	10.8	11.1^ l^	3.8
2014	40.1	30.7^ l^	9.9	13.9	4.3
2018	37.1	31.3	10.5	16.7^ h^	4.4
2022	33.7^ l^	37^ h^	6.6^ l^	17.2^ h^	5.6^ h^
Girls:					
2002	40.6^ h^	19.3	13.2^ h^	18.9^ l^	8^ l^
2006	38.3^ h^	18.9	11^ h^	22.5^ l^	9.3^ l^
2010	38.4^ h^	18.9	10.2	23.3^ l^	9.2^ l^
2014	32.9	18.5	8.2	29.4	11
2018	28.3^ l^	17.9	7.8^ l^	34.9^ h^	11.1
2022	21.9^ l^	15^ l^	5.6^ l^	40.5 ^h^	17^ h^

^h^ = significantly high value and ^l^ = significantly low value of the adjusted standardized residuals

Boys: χ^2^(*N* = 23959, *df* = 20) = 351.19, *p* < .001, Cramer’s V = 0.06

Girls: χ^2^(*N* = 24562, *df* = 20) = 1075.99, *p* < .001, Cramer’s V = 0.11

As illustrated in Fig. 1, the two most prevalent mental health profiles among boys were those of adequate mental health and perceived good health. Of these two profiles, the adequate mental health profile exhibited the highest prevalence, although this declined in the last three survey years. Additionally, there was an increase in the high psychosomatic profile from 2010 to 2022.

In contrast, the picture was different for girls, as shown in Fig. 1. Prior to 2010, adequate mental health was the most prevalent profile for girls, as it was for boys. From that year onwards, the prevalence of adequate mental health decreased, while that of high psychosomatic complaints increased for girls. Consequently, high psychosomatic complaints surpassed adequate mental health as the most common mental health profile for girls in 2018 and 2022. Another notable gender difference is that perceived good health was considerably more prevalent among boys than girls. While perceived good health increased among boys from 2018 to 2022, perceived good health decreased among girls over the same period. Perceived poor health with few signs of psychosomatic complaints was observed in approximately one in ten boys and girls, and for both sexes, perceived poor health decreased over the years.

In summary, the most striking finding between 2002 and 2022 is the sharp increase in high psychosomatic complaints among girls from 2010 to 2022, which has resulted in psychosomatic complaints becoming the most common mental health profile for girls. It should be noted that there has also been a longer-term increase in psychosomatic complaints among boys from 2010, although this increase is smaller than for girls. In addition, there was a decline in adequate health from 2010 to 2022 for both boys and girls. Finally, we compared the mental health profiles between 2002 and 2022 as effect sizes for boys and girls separately. The h-values for each mental health profile for girls were 0.41, 0.11, 0.27, -0.48, and -0.27, indicating that the effect sizes were almost large for psychosomatic complaints, slightly smaller for adequate mental health, moderate for perceived poor health, and moderate for dual health problems. The corresponding h-values for boys were lower: 0.15, -0.09, 0.20, -0.27, and -0.15. There was a moderate effect size for psychosomatic complaints and small effect sizes for the other mental health profiles. A final observation is that more girls than boys belonged to the dual health problem profile, and that this profile slowly increased over time, and more so for girls.

It is of interest to ascertain whether the changes in mental health profiles over the survey years differ between countries. To this end, separate ANOVAs were run for each gender, with the country variable included as a covariate. For boys, the country variable was not significant: F (1, 23,958) = 3.71, p = 0.054. In contrast, for girls, the country variable was significant: The analysis revealed a statistically significant difference between the survey years (F (1, 24,561) = 13.31, *p* < 0.001), bur with a low eta^2^ of 0.01. This indicates that the observed differences between the mental health profiles across the survey years were relatively consistent across countries both for boys and girls.

### Psychological and social characteristics of adolescents in the mental health profiles in 2002 and 2022

Before reporting differences between the mental health profiles for psychological and social adjustment characteristics, we examined the correlations between these adjustment characteristics and psychosomatic complaints and perceived poor overall health, aggregated across countries and survey years. We also included the measure of low life satisfaction. The results are shown in Table 8.

**Table 8:** Associations between the measures used for comparison and psychosomatic complaints, poor overall health, and low life satisfaction. The correlations are calculated across countries and survey years

	Psychosomatic complaints	Poor overall health	Low life satisfaction	Psychosomatic vs. poor health		Psychosomatic vs. low life satisfaction	
Felt stressed	.48	.36	.44	11.07	<.001	4.63	<.001
Felt lonely	.52	.38	.50	12.75	<.001	2.43	.015
Regular health habits	-.20	-.35	-.26	30.31	<.001	13.57	<.001
Good parent communication	-.26	-.24	-.35	-3.02	.003	21.70	<.001
Like school	-.20	-.21	-.29	1.60	.110	20.58	<.001
Taking medication	.28	.20	.18	3.58	<.001	6.00	<.001
Family is well off	-.13	-.21	-.26	15.38	<.001	25.60	<.001

^1^Tests of dependent correlations (two dependent groups with overlapping correlations). Steiger’s modification of Dunn & Clarke’s Z

As illustrated in Table 8, the three mental health indicators exhibited a correlation with the adjustment indicators at a comparable level. Given the considerable sample sizes for the majority of the measures, the magnitudes of the observed correlations exhibited a highly significant difference. These differences, however, can be considered small. We conclude that psychosomatic complaints have about the same associations with young people's psychological and social adjustment problems as perceived poor health and low life satisfaction. It should be added that perceived poor health and low life satisfaction had a somewhat stronger negative correlation with family affluence than did psychosomatic complaints. The correlations between psychosomatic complaints and perceived poor health and low life satisfaction were 0.36 and 0.48, respectively, when aggregated across countries and survey years. The correlation between perceived poor health and low life satisfaction was 0.42. All three correlations were significant at the level of 0.001. They cannot be considered bipolar, but rather bivariate.

How do the mental health profiles relate to the comparison variables? Table 9 shows the differences between the five mental health profiles for the measures of psychological and social adjustment problems, separately for boys and girls. The strongest differences between the mental health profiles occurred for feeling stressed and feeling lonely, with strong effect sizes for girls. For the other psychological and social adjustment measures, the effect sizes were mostly moderate for the sexes when comparing the five mental health profiles.

**Table 9:** Differences in comparison measures between the mental health profiles, calculated separately for boys and girls

	Adequate mental health	Perceived good mental health	Perceived poor health	High psychosomatic complaints	Dual health problems	Eta^2^
Feeling stressed:						
Boys	-0.06^d^	-0.32^e^	0.25^c^	0.39^b^	0.97^a^	0.12
Girls	-0.46^e^	-0.75^d^	0.15^b^	0.20^b^	0.74^a^	0.24
Feel lonely:						
Boys	-0.07^d^	-0.37^e^	0.30^c^	0.49^b^	1.11^a^	0.17
Girls	-0.40^d^	-0.72^e^	0.03^c^	0.20^b^	0.68^a^	0.21
Regular health habits:						
Boys	-0.05^c^	0.27^d^	-0.38^b^	-0.08^c^	-0.56^a^	0.13
Girls	0.06^d^	0.32^e^	-0.24^b^	-0.03^c^	-0.42^a^	0.12
Good parent communication:						
Boys	0.00^d^	0.21^e^	-0.26^b^	-0.21^c^	-0.60^a^	0.11
Girls	0.11^d^	0.35^e^	-0.20^b^	-0.11^c^	-0.50^a^	0.08
Like school:						
Boys	0.03^d^	0.23^e^	-0.25^c^	-0.36^b^	-0.81^a^	0.07
Girls	0.22^d^	0.42^e^	-0.10^c^	-0.18^b^	-0.63^a^	0.11
Taking medication:						
Boys	-0.10^c^	-0.10^c^	0.29^b^	0.20^b^	0.46^a^	0.13
Girls	-0.21^c^	-0.30^d^	0.16^b^	0.03^b^	0.46^a^	0.06
Family is well off:						
Boys	-0.03^c^	0.22^d^	-0.31^b^	-0.07^c^	-0.51^a^	0.04
Girls	0.01^c^	0.29^d^	-0.26^b^	0.05^c^	-0.41^a^	0.04

Comparison measures are transformed into Z-scores

There are significant differences, at the .001 level, in all comparisons for both boys and girls. Hence, eta^2^, as a measure of effect size, is reported

eta^2^ value .01 is small, .06 is medium, and .14 is large effect size

Different superscripts ^a b c^ represent significant differences (*p* < .05) between the four cluster groups employing Student Neuman Keul’s post-hoc test. The Dual health problems group is consistently designated superscript ^a^

It appears that adolescents with the dual health problem profile are at the highest risk for psychological and social adjustment problems. Without exception, these adolescents differ significantly from all other mental health profiles. The two other risk profiles—adolescents who perceive themselves as having poor health and adolescents with high psychosomatic complaints—differ significantly from adolescents with adequate mental health and adolescents who perceive themselves as having good health on all comparison variables, but they do not differ much from each other.

In recent decades, an increase in psychosomatic problems has been identified as a particular indicator of deteriorating mental health among adolescents, with a greater prevalence observed among girls. However, the present results suggest that this conclusion should be qualified. Adolescents in the high psychosomatic profile did indeed show poorer psychological and social adjustment than those in the first two profiles (adequate health and perceived good health). However, their psychological and social adjustment problems were little different from those of adolescents in the perceived poor health profile, and their adjustment problems were considerably lower than those of adolescents in the dual health profile.

Finally, it should be added that the lowest level of perceived family wealth was found in the dual health profile. Adolescents in the perceived poor health profile also had low scores on the measure of family affluence, whereas the family affluence of adolescents in the high psychosomatic complaints profile was comparable to the average.

Overall, the most striking finding in Table 9 is that adolescents who experience high levels of psychosomatic problems and perceive themselves to be in poor health have low levels of psychological and social adjustment. These adolescents represent the primary at-risk group.

### Robustness of the mental health profiles

The perceived overall health of an individual has been utilized as one of the two indicators of mental health in all cluster analyses. In order to test the robustness of the mental health profiles, we exchanged perceived overall health with a measure of adolescents' subjective well-being, life satisfaction. This latter measure also shows a lack of change over the survey years.

A new cluster analysis was conducted across countries and survey years with low life satisfaction and psychosomatic complaints as the two cluster variables (see Table 3 for comparison). The results are presented in Table 10. The five mental health profiles presented in Table 10 are very similar to those previously reported in Table 3. The proportions of individuals in each profile and the gender differences are comparable. The two clusters were cross-tabulated (χ2 (16 df) = 42333, *p* < 0.001). The contingency coefficient was 0.68, and Cramer's V was 0.47, indicating that the two cluster solutions were moderately associated. It can be concluded that the same mental health profiles appear robustly as cluster constellations when exchanging perceived overall health with adolescents' perceptions of low life satisfaction.

**Table 10:** Cluster analyses of psychosomatic complaints and low life satisfaction in 2018. The analyses are conducted across countries (*N* = 6460)

	Adequate mental health	Perceived good health	Perceived poor health	High psychosomatic complaints	Dual health problems
Psychosomatic complaints	0.10	-0.91	-0.14	1.36	1.66
Low life satisfaction	-0.33	-0.65	1.15	0.07	2.06
N	13408	17951	6202	6701	4075
%	27.7	37.1	12.8	13.9	8.4
% girls^1^	52.5	36.6	51.9	70.3	73.5

Low value is < -0.70, average value is between -0.70 and 0.70, high value is > 0.70

The five clusters explained 75 percent of the total error sums of squares

^1^ Chi^2^ (4 df) = 3320.57 *p* < .001, Cramer’s V = 0.26

Table 11 presents a comparison between the mental health profiles for the psychological and social adjustment measures, with life satisfaction included as a clustering variable. The results are largely consistent with those reported in Table 10. Effect sizes were generally modest when the five mental health profiles were compared for the measures. Adolescents in the dual health profile exhibited significant differences from the other mental health profiles for all comparison measures. Adolescents in the perceived poor health and the high psychosocial complaints did not differ between themselves but showed significantly worse adjustment than the adolescents in the adequate mental health and perceived good mental health profiles. The lowest level of perceived family wealth was found in the dual health profile. Overall, it appears that the results are very similar when exchanging the clustering variable perceived poor health with low life satisfaction.

**Table 11:** Differences in comparison measures between mental health profiles using psychosomatic complaints and low life satisfaction as clustering variables

	Adequate mental health	Perceived good mental health	Perceived poor health	High psychosomatic complaints	Dual health problems	Eta^2^
Feel stressed	0.06^d^	-0.32^e^	0.25^c^	0.39^b^	0.97^a^	0.12
Feel lonely	-0.07^d^	-0.37^e^	0.30^c^	0.49^b^	1.11^a^	0.17
Regular health habits	-0.05^c^	0.27^d^	-0.38^b^	-0.08^c^	-0.56^a^	0.13
Good parent communication	0.00^d^	0.21^e^	-0.26^b^	-0.21^c^	-0.60^a^	0.11
Like school	0.03^d^	0.23^e^	-0.25^c^	-0.36^b^	-0.81^a^	0.07
Taking medication	-0.10^c^	-0.10^c^	0.29^b^	0.20^b^	0.46^a^	0.13
Family is well off	-0.03^c^	0.22^d^	-0.31^b^	-0.07^c^	-0.51^a^	0.04

Comparison measures are transformed into Z-scores

There are significant differences, at the .001 level, in all comparisons. Hence, eta^2^, as a measure of effect size, is reported

eta^2^ value .01 is small, .06 is medium, and .14 is large effect size

Different superscripts ^a b c^ represent significant differences (*p* < .05) between the four cluster groups employing Student Neuman Keul’s post-hoc test. The Dual health problems group is consistently designated superscript ^a^

## Discussion

The objective of this study was to examine the similarities in longer-term trends in mental health indicators among adolescents in five Nordic countries: Denmark, Finland, Iceland, Norway, and Sweden. To circumvent the conventional bipolar model of mental health in the literature, in which a singular dimension is employed to delineate mental health, ranging from good mental health (or the absence of complaints) to mental health issues (manifested by a high prevalence of psychopathological complaints), the dual-factor model was an attempt combine non-clinical mental health indicators, namely perceived health status and manifest psychosomatic complaints, in a single bivariate but not necessarily bidirectional mental health map [7–9, 53]. A cluster analysis was conducted using perceived overall health and psychosomatic complaints as clustering variables. First, the results revealed that the same mental health profiles were observed in all Nordic countries, including those with adequate, perceived good, perceived poor, high psychosomatic complaints, and dual health problems (both high psychosomatic complaints and perceived poor health). Second, the percentage of respondents in each of the profiles did not differ substantially between the Nordic countries. Third, the results demonstrated comparable changes over time, from 2002 to 2022, in the mental health profiles across countries. In terms of differences between survey years, the adequate mental health profile and the perceived poor health profile decreased between 2002 and 2022, while the high psychosomatic complaints profile and the dual health problems profile increased over the same 20 years. Consequently, it can be concluded that the mental health profiles among adolescents in the five different countries represent a general developmental pattern in the Nordic region.

The present study builds directly on two recent studies that applied the dual-factor model in one of these Nordic countries. In one study [42], a cluster analysis of perceived overall health and psychosomatic complaints among 11-, 13-, and 15-year-old Swedish children and adolescents found the same mental health profiles in each age group (four cluster were extracted, in contrast to five in the present study). Therefore, not only do distinct mental health profiles of 15-year-old Nordic adolescents align; they are also found in earlier ages. In the other study [26], the same analysis of time trends for mental health indicators as used here was employed for analyses from 2002 to 2022 for Swedish 15-year-old adolescents. The same mental health profiles were found in all survey years. The present study extends these analyses to five Nordic countries. It can be concluded that the observed trends in Sweden are not unique to Swedish conditions. The time trends for these mental health profiles are similar to the time trends for the same mental health profiles in the other Nordic countries.

The finding of similar trends in the five countries suggests that explanations should be sought in conditions that are common to the countries rather than specific to each country. Indeed, the Nordic countries—Denmark, Finland, Iceland, Norway, and Sweden—are geographically close, share many cultural and value similarities and are welfare states with a high standard of living, a strong emphasis on education and gender equality, universal health care and public spending on welfare and public health promotion [54, 55]. However, country-specific explanations for changes over time in mental health indicators are not uncommon. For instance, the Swedish Public Health Agency has concluded that there has been a notable decline in mental health among Swedish 15-year-old adolescents based on observed trends in psychosomatic complaints. The increase over time in this mental health indicator was attributed primarily to stress experienced at school due to perceived shortcomings in the Swedish education system and the characteristics of the Swedish labor market [27, 56]. These conclusions were derived from an analysis of temporal trends in psychosocial problems among Swedish adolescents only. If the assumptions are accurate, it can be reasonably assumed that these explanatory factors will also apply to other Nordic samples. The distinction between country-specific and country-general explanations has implications for the research strategies of future studies that attempt to explain changes in mental health across geographically close countries that share many cultural and societal similarities and values.

The majority of the observed changes in mental health profiles occurred gradually over time, with a notable decrease in the proportion of individuals with an adequate mental health profile from 2002 to 2022, accompanied by a more pronounced increase in the proportion of individuals with a high psychosomatic complaints and a dual health profile. A comparison of the years 2002 and 2022 revealed a decrease in the adequate mental health and perceived poor health profiles, with a moderate effect size, and an increase in the high psychosomatic complaints and dual health problems profiles, also with a moderate effect size. The association between year differences and mental health profiles is at most moderate. In terms of practical implications, however, the differences in the prevalence of mental health problems between 2002 and 2022 are substantial. Not only has the proportion of 15-year-olds with high psychosomatic problems and dual health problem profiles more than doubled over this period, but the proportion of young people with an adequate health profile has fallen by 30 percent. This information is more specific than that presented in earlier studies, which only indicated that psychosomatic problems among young people have more than doubled in recent decades |27]. It can be concluded that, under certain conditions, person-oriented methods can provide more specific information about changes over time than variable-oriented methods [57].

### Sex differences

Compared to the more limited differences between the five Nordic countries for the mental health profiles over the survey years, the sex differences were more pronounced. Girls were underrepresented in the adequate mental health and perceived good health profiles and overrepresented in the high psychosomatic and dual health problem profiles.

The most striking gender differences concern the changes in the individual mental health profiles of boys and girls over the survey years. There were relatively small changes in the profile trends for boys. The two mental health profiles that were most common among boys from 2002 to 2022 were adequate health and perceived good health. The only profile that showed more significant changes over time for them was the increase in psychosomatic complaints. The changes were more pronounced for girls. There was an increase in psychosomatic complaints with an almost large effect size when comparing 2002 to 2022. This increase, together with a decrease in adequate mental health, meant that whereas at the beginning adequate mental health was the most common mental health problem, over time this changed to a situation where the most common mental health profile was high psychosomatic problems for girls. This shift over time is an important finding of this study. The high psychosomatic complaints profile is the most common mental health profile for girls from 2018 onwards.

The profile with dual health problems—adolescents with high levels of psychosomatic problems and high levels of perceived unhealthiness—accounted for about 8 per cent of all adolescents in all countries and survey years. Dual health problems were much more common among girls than among boys. This difference, with two to three times as many girls as boys in the profile of dual health problems, was as true in 2002 (2.7 per cent boys and 8 per cent girls) as it was in 2022 (5.6 per cent boys and 17 per cent girls). This means that the sex differences that were observed in 2022 already existed twenty years earlier, in 2002.

### Three At-risk profiles

#### High psychosomatic complaints and perception of poor health

The increase in psychosomatic complaints over many years has been reported in separate analyses of the five Nordic countries and in studies combining national samples from several of these countries. The changes over the years have been taken as a strong sign that mental health problems are increasing among adolescents, especially among girls [2–5, 58]. Our analyses show that this increase is not limited to girls. It is also evident among boys. The present analyses pinpoint the time when the psychosomatic problems started to increase among both boys and girls in the Nordic countries. After smaller changes between 2002 and 2010, there was a larger increase for both sexes between 2010 and 2022, with girls showing a larger increase than boys in the latter years. In 2010, 11 percent of boys and 23 percent of girls were in the high profile of psychosomatic complaints. By 2022, this had increased to 17 percent for boys and 41 percent for girls. In terms of effect size, this difference between the sexes is small in both 2010 and 2022, but compared with the changes in the other profiles, the increase in this profile for boys was greater than for any of the other mental health profiles between 2002 and 2022, and the increase in the high psychosomatic complaints profile for girls meant that by 2018 and 2022 the high psychosomatic complaints profile was the most common mental health profile, more common than the adequate health profile. Given these time trends, it is important to have a more nuanced understanding of the role that psychosomatic problems play in mental health.

The high psychosomatic complaints profile is not the only 'risk' profile. Perceived poor overall health and dual health problems—defined as high psychosomatic complaints in addition to perceived poor health—are the other two risk profiles. There is an obvious difference in the time trend between the profile of high psychosomatic complaints and the profile of young people who perceived their health as poor. Between 2002 and 2022, the proportion of people with the perceived poor health profile in the Nordic countries fell by more than 50 per cent, from 12.8 to 6.1 per cent, while the high psychosomatic complaints profile more than doubled, from 13.6 to 28.8 per cent.

Adolescents in the high psychosomatic profile and the perceived poor overall health profiles exhibited comparable levels of psychological and social adjustment problems. For some maladjustment indicators, the problems were more pronounced in the high psychosomatic profile, while for other indicators, the opposite was true. Their levels of problems were greater than those observed in the two positive mental health groups, but they were less severe than those observed in adolescents in the dual health profile.

The existing literature has not devoted significant attention to young people who perceive themselves to be in poor health but who do not exhibit elevated levels of psychosomatic symptoms. The measures of perceived poor health status and psychosomatic complaints demonstrated comparable levels of correlation with the measures of poor psychological and social adjustment. There is a substantial scope for further investigation into the adolescents who fall within the perceived poor health profile. Further research is required to ascertain the reasons for the significant decline in the proportion of adolescents in the perceived poor health profile over the past two decades, in contrast to the observed increase in the other two at-risk groups. Additionally, it is crucial to investigate the factors that contribute to the adjustment difficulties experienced by these adolescents. Furthermore, future research should seek to understand why the perceived poor health profile comprises an equal proportion of boys and girls, whereas girls are markedly overrepresented in the other two risk profiles.

#### The dual problems profile

Based on the assumption of heterogeneity among adolescents with high psychosomatic complaints in two earlier studies, we hypothesized that two distinct mental health profiles would emerge: one comprising adolescents with high psychosomatic complaints and an adequate perceived health status, and another group with high psychosomatic complaints and a perceived poor overall health status. This distinction was subsequently validated. Of the adolescents with these two mental health profiles, 73% reported high psychosomatic complaints and an average level of perceived poor health, while the remaining 27% reported both high psychosomatic complaints and high perceived poor health (Table 4). The two at-risk groups exhibited similar characteristics, including an overrepresentation of girls (68.9% in the first profile and 72.9% in the second) and a doubling of the proportions between 2002 and 2022. The proportion of individuals in the first group exhibiting these characteristics increased from 13.6% in 2002 to 28.8% in 2022 (see Table 5). The proportion of individuals in the second group exhibiting these characteristics increased from 5.4% in 2002 to 11.8% in 2022. This may be the point at which the two profiles’ similarities end, because they appear to be fundamentally different in other respects. The profile of adolescents with high psychosomatic problems and perception of being overall unhealthy includes adolescents who have considerably more adjustment problems than the profile of adolescents who also have high psychosomatic problems but who do not perceive their overall health to be different from that of the average person. A comparison of their psychological and social adjustment confirms this. This finding may help to explain the discrepancy between mental health symptoms and perceived general health that has been reported in previous studies [59].

A comparison of the five mental health profiles for the measures of psychological and social adjustment problems has revealed that the dual health problems profile represents the most significant risk profile. For all the adjustment problems examined, a significant difference was observed between this profile and the other two at-risk profiles. This indication of risk applies to both boys and girls in the dual health problems profile. Thus, of the three risk profiles—perceived poor health, high psychosomatic complaints, and dual health problems—the dual health problems profile represents the most significant risk profile.

Previous claims that an increase in psychosomatic problems over the last decades is strong evidence of a rapid rise in mental health problems among young people may be unfounded. While psychosomatic problems among adolescents have increased considerably since 2010, the psychological and social problems that accompany high levels of psychosomatic complaints do not appear to be as extensive as previously thought. Adolescents in the high psychosomatic complaints profile exhibit significantly greater but not substantially greater psychological and social adjustment problems than adolescents in the two good mental health groups. Consequently, an increase in high psychosomatic symptoms among adolescents over time does not necessarily indicate that they experience profoundly higher levels of psychological and social problems. It is possible that the mental health literature, which uses psychosomatic complaints as the strong indicator of increasing mental health problems among Nordic adolescents, may have reached premature conclusions. A more accurate representation of the time trends in problematic mental health among adolescents may be the increase in dual health problems from 5.4 to 11.8 percent between 2002 and 2022, rather than the increase in high psychosomatic complaints from 13.6 to 28.8 percent (and even from 18.9 to 49.5 percent for girls). A further comment is warranted here. A multitude of studies have demonstrated an increase in psychosocial symptoms among young people in the Nordic countries over the past three decades, leading to a conclusion that there has been a decline in mental health [27, 59]. However, it is crucial to acknowledge that adolescents’ perceived health status and life satisfaction have remained relatively stable over time. Consequently, it seems inadvisable to rely on a single measure in reaching conclusions about the development over time of young people’s mental health.

Finally, in order to differentiate adolescents with high psychosomatic symptoms, we have used these adolescents’ perception of their poor health status, which ranged from poor to excellent. It should be noted that other health indicators are possible, and in a robustness analysis, we examined the adolescents’ low life satisfaction. A cluster analysis conducted across all countries yielded similar five mental health profiles as when perceived poor health status was employed as the clustering variable. Moreover, analogous findings were observed when comparing the mental health profiles derived for the psychological and social adjustment measures. It can be concluded that conceptually similar constructs for Nordic adolescents’ perception of their health and well-being differentiate adolescents with high psychosomatic problems into two distinct groups: those with more mild and more severe adjustment problems.

### Strengths and limitations

In numerous previous studies on changes in adolescents’ mental health over time, time trends for different mental health indicators have been reported separately. Conclusions about these changes have been primarily based on changes over time in one indicator, psychosomatic symptoms. This study’s primary strength lies in its use of an analytical framework, the dual-factor model, that combines relevant mental health indicators. This framework enables a more comprehensive understanding of the evolution of mental health among 15-year-old adolescents in the Nordic countries between 2002 and 2022.

Another major strength of the study is that it is based on large-scale surveys of nationally representative samples of 15-year-olds in Denmark, Finland, Iceland, Norway, and Sweden. The standardized international research protocol of the HBSC study includes a careful process of translation-back-translation of questions to be used in data collection in different countries, as well as measures to ensure consistency in survey instruments, data collection and processing procedures. The measures used have good validity and reliability according to previous research [45–51].

Repeated surveys are needed to identify stability and change in adolescent health over time. However, the use of a cross-sectional design has a weakness in that it does not allow causal inferences to be made. In addition, questions asked only of adolescents do not allow for the inclusion of independent sources of information from parents, teachers and friends. Perhaps most importantly, it is unknown whether the significant others of these young people are aware of the mental health problems that some of them are experiencing.

## Conclusion

A person-oriented approach identified five distinct mental health profiles among adolescents in all Nordic countries and similar changes over time, from 2002 to 2022, for these profiles. These profiles were based on psychosomatic symptoms and perceived overall health. The findings have implications for understanding the developmental trajectory of mental health over longer periods of time. It has been proposed in numerous publications that the increase in psychosomatic symptoms over longer periods of time is evidence that the mental health among adolescents has deteriorated significantly over the past three decades in the Nordic countries. However, a focus on psychosomatic complaints alone when trying to understand mental health among adolescents may not be sufficient. Adolescents with high psychosomatic complaints do not constitute a homogeneous group. Some of them have significant psychological and social adjustment issues, while others have less problematic circumstances. The findings from combined analyses of psychosomatic symptoms and perceived overall health indicate that when adolescents with high psychosocial problems also perceive themselves to have poor overall health, it is indicative of more severe mental health problems. Conversely, when they do not, it is indicative of less severe adjustment problems.

## Data Availability

The data presented in this study are available on reasonable request from the first author or from HBSC Data Management Centre, University of Bergen, Norway. Data from the three latest data collection can also be explored from the HBSC study data browser: https://data-browser.hbsc.org/.

## Abbreviations

HBSC: Health Behaviour in School-aged Children
MUCF: Swedish Agency for Youth and Civil Society
HBSC-SCL: Health Behaviour in School-aged Children- Symptom Check Complaint List
WHO/EURO: European region of World Health Organization
ANOVA: Analysis of Variance
FORTE: : Swedish Research Council for Health, Working Life and Welfare
