# Correction to: Mental health profiles of 15-year-old adolescents in the Nordic Countries from 2002 to 2022: person-oriented analyses

**DOI:** 10.1186/s12889-024-20218-0

**Published:** 2024-10-07

**Authors:** Charli Eriksson, Håkan Stattin

**Affiliations:** 1 Department of Learning, Informatics, Management and Ethics, Karolinska Institute, Stockholm, SE‑17177 Sweden; 2 Department of Psychology, Uppsala University, Box 1225, Uppsala, 75142 Sweden

**Correction to: BMC Public Health 24, 2358 (2024)**

10.1186/s12889-024-19822-x

In the original publication of this article an error was introduced during the publication process which affected the superscript numbers of the first column in Table 2. The incorrect and correct Table 2 are shown in this correction article, the original article has been updated. The publisher apologizes for the inconvenience caused to the authors & readers.

**Incorrect** Table 2

**Table 2:** Trends in perceived low overall health, low life satisfaction and psychosomatic complaints for the five countries

	Perceived low overall health	Low life satisfaction	Psychosomatic complaints
2002	-0.01^a^	-0.01^b^	-0.27^e^
2006	-0.01^a^	-0.08^c^	-0.14^d^
2010	-0.00^a^	-0.06^c^	-0.13^d^
2014	-0.01^a^	0.02^b^	-0.00^c^
2018	-0.00^a^	0.01^b^	0.23^b^
2022	0.02^a^	0.13^a^	0.32^a^

The measures are standardized

Different superscripts ^a b c^ represent significant differences (*p* < .05) between the four cluster groups employing Student Neuman Keul’s post-hoc test

Perceived low overall health: F (5, 48875) = 0.74, *p* = .592, eta2 = 0.00 Low life satisfaction: F (5, 48395) = 50.50, *p* < .001, eta2 = 0.01

Psychosomatic complaints: F (5, 48810) = 390.33, *p* < .001, eta2 = 0.04

**Correct** Table 2

**Table 2:** Trends in perceived low overall health, low life satisfaction and psychosomatic complaints for the five countries

	Perceived low overall health	Low life satisfaction	Psychosomatic complaints
2002	-0.01^a^	-0.01^b^	-0.27^e^
2006	-0.01^b^	-0.08^c^	-0.14^d^
2010	-0.00^b^	-0.06^c^	-0.13^d^
2014	-0.01^a^	0.02^b^	-0.00^c^
2018	-0.00^a^	0.01^b^	0.23^b^
2022	0.02^a^	0.13^a^	0.32^a^

The measures are standardized

Different superscripts ^a b c^ represent significant differences (*p* < .05) between the four cluster groups employing Student Neuman Keul’s post-hoc test

Perceived low overall health: F (5, 48875) = 0.74, *p* = .592, eta2 = 0.00 Low life satisfaction: F (5, 48395) = 50.50, *p* < .001, eta2 = 0.01

Psychosomatic complaints: F (5, 48810) = 390.33, *p* < .001, eta2 = 0.04

